# Pneumonitis after radiotherapy for lung cancer (PARALUC): an interventional study to create a symptom-based scoring system for identification of patients developing radiation pneumonitis

**DOI:** 10.1186/s12885-020-07291-5

**Published:** 2020-08-20

**Authors:** Dirk Rades, Elisa Marie Werner, Esther Glatzel, Marie-Christine Eggert, Denise Olbrich, Soeren Tvilsted, Sabine Bohnet

**Affiliations:** 1 Department of Radiation Oncology, University of Lübeck, Ratzeburger Allee 160, 23562 Lübeck, Germany; 2 Centre for Clinical Trials Lübeck, Lübeck, Germany; 3 Research Projects and Clinical Optimization, Zealand University Hospital, Koege, Denmark; 4 Department of Pulmonology, University of Lübeck, Lübeck, Germany

**Keywords:** Lung cancer, Radiotherapy, Radiation pneumonitis, Symptom-based score, Prevalence

## Abstract

**Background:**

Pneumonitis is a possible side effect of radiotherapy for lung cancer. Since it can occur up to several months following treatment, symptoms may not be associated with previous radiotherapy, and pneumonitis can become severe before diagnosed. This study aimed to develop a symptom-based scoring system to contribute to earlier detection of radiation pneumonitis requiring medical intervention (grade ≥ 2).

**Methods:**

Patients irradiated for lung cancer complete a paper-based questionnaire (symptom-based score) during and up to 24 weeks following radiotherapy. Patients rate symptoms potentially associated with pneumonitis, and scoring points are assigned to severity of these symptoms. Sum scores are used to identify radiation pneumonitis. If radiation pneumonitis is suspected, patients undergo standard diagnostic procedures. If grade ≥ 2 pneumonitis is confirmed, medical intervention is indicated. The discriminative power of the score will be assessed by calculating the area under the receiver operating characteristic curve (AUC). If statistical significance of the AUC is reached, the optimal sum score to predict radiation pneumonitis will be established, which is defined as a cut-off value with sensitivity ≥90% and specificity ≥80%. Assuming a ratio between patients without and with pneumonitis of 3.63, a sample size of 93 patients is required in the full analysis set to yield statistical significance at the level of 5% with a power of 90% if the AUC under the alternative hypothesis is at least 0.9. Considering potential drop-outs, 98 patients should be recruited. If > 20% of patients are not satisfied with the score, modification is required. If the dissatisfaction rate is > 40%, the score is considered not useful. In 10 patients, functionality of a mobile application will be tested in addition to the paper-based questionnaire.

**Discussion:**

If an optimal cut-off score resulting in sufficiently high sensitivity and specificity can be identified and the development of a symptom-based scoring system is successful, this tool will contribute to better identification of patients experiencing pneumonitis after radiotherapy for lung cancer.

**Trial registration:**

Clinicaltrials.gov (NCT04335409); registered on 2nd of April, 2020.

## Background

Lung cancer belongs to the most common types of solid cancer in Europe and Northern America [1]. Most patients with small-cell lung cancer (SCLC) receive radiotherapy in combination with chemotherapy as definitive treatment [2]. Also, a considerable number of patients with advanced non-small-cell lung cancer (NSCLC) are treated with radiotherapy with or without concurrent chemotherapy [2]. Radiation pneumonitis is a possible side effect of radiotherapy for lung cancer. Severe pneumonitis was reported to be fatal in approximately 2% of patients experiencing this adverse event [3].

In our centre, the prevalence of symptomatic radiation pneumonitis was 7.6% in patients irradiated for lung cancer [4]. Moreover, risk factors for radiation pneumonitis were identified including a mean radiation dose to the ipsilateral lung of > 20 Gy or a mean dose of > 13 Gy plus at least one other factor such as significant cardiovascular disease, history of heavy smoking (≥40 pack years), and systemic treatment (chemotherapy or immunotherapy) prior to or during radiotherapy [4]. The prevalence of symptomatic radiation pneumonitis in patients with such risk factors treated between 2016 and 2018 was 18.8%.

Pneumonitis can occur up to 23 weeks following radiotherapy [4, 5]. Therefore, the symptoms may not be associated with previous radiotherapy, and pneumonitis may be missed [5]. It would be important to identify patients developing radiation pneumonitis and requiring medical treatment more early. This study aims to develop a symptom-based scoring system that contributes to an earlier detection of radiation pneumonitis requiring medical intervention (grade ≥ 2) after radiotherapy for lung cancer [6]. This scoring system is a prerequisite for a mobile application, which can be used by the patients at home to rate their symptoms possibly related to pneumonitis.

## Methods and design

This is a single-centre and single-arm prospective interventional study performed in an academic hospital (university medical centre), which aims to assess the performance of a new symptom-based score and to identify its optimal scoring point for detection of patients developing pneumonitis following radiotherapy for lung cancer.

### Objectives and endpoints

The main goal of this trial is to establish the performance characteristics and to develop a decision-algorithm of a new symptom-based scoring system with respect to the identification of patients developing pneumonitis after radiotherapy of lung cancer. Following end of study, the patients receive the standard follow-up program for lung cancer patients. Harm from trial participation is not expected, since all participating patients receive the same anticancer treatment as they would have received if not participating.

Primary endpoint (outcome): To assess the performance characteristics of the symptom-based scoring system for detection of radiation pneumonitis the receiver operating characteristic (ROC) curve is used to show the connection between sensitivity and specificity for every possible cut-off for the scoring system and to select the optimal scoring point for detection of radiation pneumonitis. The area under the ROC curve (AUC) is calculated to prove the diagnostic ability of the scoring system.

In addition, the following endpoints (outcomes) will be evaluated:

1. Positive and negative predictive values associated with each point of the symptom-based scoring system.
2. Patient satisfaction with the symptom-based scoring system (symptom-questionnaire, paper version), assessed at the end of radiotherapy.
3. Quality of life: Evaluation prior to radiotherapy, at the end of radiotherapy and at the end of follow up using the EORTC QLQ-C30 Version 3.0 and the EORTC QLQ-LC13 (https://qol.eortc.org). Both quality of life scores will be correlated to the scoring points of the symptom score.
4. Patient satisfaction with a mobile application that asks the same questions as the paper-based symptom-questionnaire (10 patients), assessed at the end of radiotherapy.

### Eligibility criteria

#### Inclusion criteria

1. Histologically proven lung cancer.
2. Indication for radiotherapy.
3. Risk factors for developing radiation pneumonitis.
4. Age ≥ 18 years.
5. Written informed consent.
6. Capacity of the patient to cooperate.

Informed consent will be taken by specially trained physicians registered as investigators for this trial. Risk factors include mean dose to ipsilateral lung > 20 Gy or mean dose > 13 Gy plus at least one other factor (significant cardiovascular disease, history of heavy smoking (≥40 pack years), chemotherapy or immunotherapy) [4, 7–13].

#### Exclusion criteria

1. Pregnancy, Lactation.
2. Limited legal capacity or being under legal supervision.
3. Baseline score of > 2 points, as these patients will likely not be able to tolerate the planned treatment including the full radiation dose.

#### Assessments

The following parameters will be recorded prior to the start of radiotherapy: medical history, concomitant diseases, concomitant medication, physical examination, demographics (age, date of birth, gender), body height and weight, performance status, primary tumour type and stage, histology, histologic grading, previous and planned cancer treatment, lung function test and quality of life.

The following parameters will be assessed during the course of the trial:

1. Symptoms of Pneumonitis
2. Quality of life will be assessed at the end of radiotherapy and at the end of the study using the EORTC QLQ-C30 version 3.0 and the EORTC QLQ-LC13 (https://qol.eortc.org).
3. Adverse events other than pneumonitis will be assessed on an ongoing basis according to CTCAE v5.0 [13]. Serious adverse events and unexpected adverse events must be reported within 24 h after their detection/onset by fax to the coordinating investigator.

**Fig. 1:**
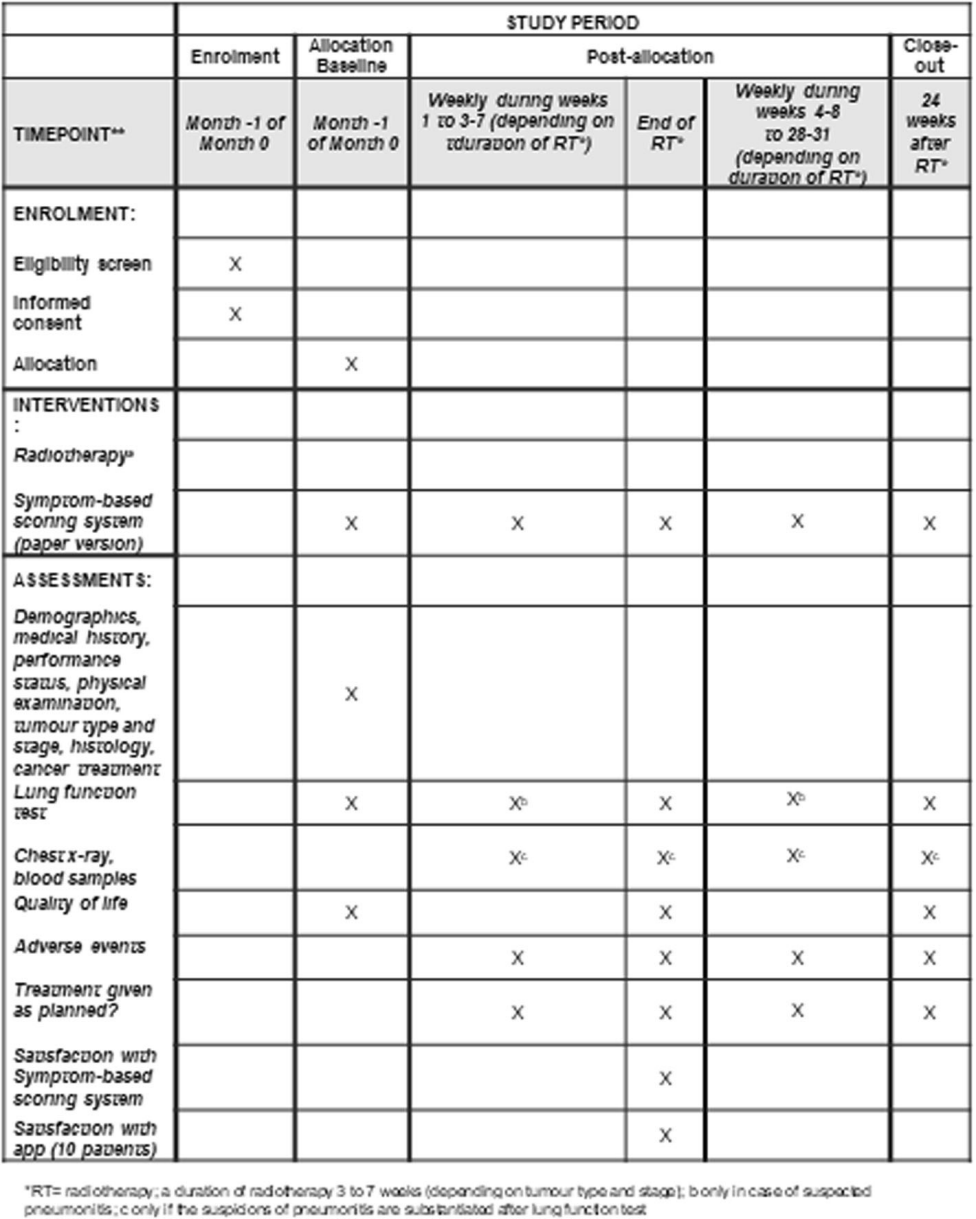
Schedule of enrolment, interventions and assessments

The timeline of the study procedures including the assessments is shown in Fig. 1.

During the period of radiotherapy, patients are seen at least 5 days per week by medical staff members. Following radiotherapy, patients are contacted every week until the end of study. Thus, it is unlikely that patients are lost to follow up. If patients withdraw their consent to participate in the trial or die during the study, the data available until this point in time are used for analyses.

### Interventions

In this study, patients receive standard radiotherapy for lung cancer [2]. Standard treatment will be modified individually and tailored to a patient’s situation if necessary the same way as it would have been done without participation in this study. If required, any type of concomitant care and interventions are permitted during the trial for treatment of other treatment-related toxicities and co-morbidities not related to radiotherapy or radio-chemotherapy.

#### Symptom-based scoring system (paper version)

The patients complete a paper-based questionnaire (symptom-based scoring system) once a week during and up to 24 weeks following radiotherapy. The patients state and score symptoms potentially associated with pneumonitis, namely cough, shortness of breath and fever. Other pneumonitis-associated symptoms that occur less frequently and are less specific have not been included in the scoring system. For example, thoracic pain is much more often related to tumour progression and, therefore, will have likely decrease the specificity of a scoring system aiming to identify pneumonitis. Scoring points are assigned to the severity of the symptoms, and the resulting sum scores are used to identify radiation pneumonitis (Table 1). During the radiotherapy period, patients complete the paper-based questionnaire prior to standard appointments with a physician. Following radiotherapy, they are contacted by phone (to minimise the number of visits to the hospital) once a week for completion of the questionnaire. In case of an increase of the total score when compared to baseline, patients receive either a follow-up telephone call after 3 days, are asked to come to the hospital as outpatients or are admitted to hospital. In case of suspected pneumonitis, patients undergo lung function tests. The suspected diagnosis of pneumonitis is considered substantiated in case of a decrease in forced expiratory volume in 1 s (FEV1) and diffusing capacity of the lung for carbon monoxide (DLCO) to less than 75% from baseline values [14, 15]. In this situation, patients receive a chest x-ray plus/minus computed tomography. Radiation pneumonitis is considered confirmed, if opacities confined to the irradiation fields are seen on chest x-ray and/or ground-glass opacities (focal or nodular), consolidation or both are seen on computed tomography [16, 17]. If the diagnosis symptomatic radiation pneumonitis (grade ≥ 2) has been confirmed, patients receive medical intervention. The vast majority of the patients receive prednisolone, which is considered the mainstay of the treatment for radiation pneumonitis [5, 6, 18]. If pneumonitis is not confirmed and symptoms are caused by other (e.g. underlying) disease, the patients receive treatment for this situation.

**Table 1:** Scoring points assigned to symptoms potentially associated with radiation pneumonitis, based on common terminology criteria for adverse events (CTCAE) v5.0 [6]

Symptom	Severity of the Symptom (as stated by the patients)	Points
**Cough**	No	0
	yes, a little/sometimes	1
	yes, moderate/regularly	2
	yes, severe/permanently	3
**Shortness of breath**	No	0
	yes, with intense exertion (e.g. climbing stairs)	1
	yes, with mild exertion (e.g. walking on flat ground)	2
	yes, at rest	3
**Fever**	No	0
	yes, between 37.6 und 38.0 °C	1
	yes, between 38.1 und 39.0 °C	2
	yes, higher than 39.0 °C	3

The symptom-based sum score is correlated to pneumonitis (yes vs. no). At the end of radiotherapy, patients are asked to complete a questionnaire (modified according to [19] (https://www.ueq-online.org) regarding their satisfaction with the score. In case of a dissatisfaction rate > 20%, the score needs modifications before it can be used in future studies. In case of a dissatisfaction rate > 40%, the symptom-based scoring system will be considered not useful.

#### Symptom-based scoring system (Mobile application)

Prior to the prospective study, 30 healthy volunteers are asked to complete a questionnaire regarding the functionality and practicability of a mobile application (app) to identify and solve relevant problems. Afterwards, the paper-based score (questionnaire) is supplemented by the app asking the same questions regarding symptoms in 10 patients. The app has been developed by a professional company. At the end of radiotherapy, the 10 patients are asked to complete a questionnaire (modified according to [19] (https://www.ueq-online.org)) regarding their satisfaction with the app. In case of a dissatisfaction rate > 20%, the app needs modifications before investigated in future studies. In case of a dissatisfaction rate > 40%, the app is considered not suitable for further investigation.

If patients withdraw their consent to participate in the trial, study-specific interventions are discontinued. If patients experience adverse events, which do not allow them to complete the questionnaires, the study-specific interventions are interrupted or, if necessary, discontinued.

### Sample size calculations

The main goal of this trial is to evaluate the usefulness of a new symptom-based scoring system for identification of patients developing pneumonitis after radiotherapy for lung cancer. The discriminative power of the symptom-based score will be assessed by calculating the area under the receiver operating characteristic (ROC) curve (AUC). The following assumptions are made:

- The two-sided significance level is set to 5%.
- Under the alternative hypothesis an AUC of 0.9 is assumed since this is decided to be an excellent diagnostic accuracy for the symptom-based scoring system worth to be considered for future routine use.
- The power to yield statistical significance is set to 90%.
- 78.4% subjects will end the study event-free, whereas 21.6% will experience radiation pneumonitis, i.e. ratio between negative and positive cases is 3.63.

Based on these assumptions above, 93 patients (20 with radiation pneumonitis and 73 without radiation pneumonitis) are required within the Full Analysis Set using a two-sided asymptotic test. The calculations were performed with MedCalc software Version 19.1.5 (MedCalc software bv, Belgium). The number of 20 patients with radiation pneumonitis is considered realistic, since the average cumulative number of events (pneumonitis) in the previous retrospective study was 0.525 per month [4]. Thus, the number of events will be 19 in 36 months. Moreover, it can be assumed that due to the weekly visits of the patients for 24 weeks following radiotherapy (instead of the standard, i.e. only one visit about 6–8 weeks following radiotherapy) and the prospective design of the present study, at least 15% more patients developing pneumonitis will be identified. Thus, 22 (19 × 1.15) events can be expected in 36 months, and the required 20 events can be expected in 33 months. Assuming that 5% of patients will not qualify for Full Analysis Set, a total of 98 patients should be recruited. The Full Analysis Set includes all patients who started radiotherapy for lung cancer. Evaluation with respect to the primary endpoint is performed in those patients, who are available for assessment and have completed at least 75% of the questionnaires (paper version) regarding the symptom-based scoring system.

All lung cancer patients at the trial centre will be screened. Recruitment of all 98 patients (93 patients plus drop-outs) should be completed within 33 months. The treatment period will be 6–7 weeks, and the follow up period 24 weeks. This equals a total running time for the trial of approximately 40 months.

### Statistical methods for primary and secondary outcomes

#### Primary endpoint

The primary aim of the study is to assess the performance characteristics of the symptom-based scoring system for detection of radiation pneumonitis. To allow for patient-based analyses, the scores documented for each patient over time will be reduced to one clinically relevant, patient-specific value only. The following pragmatic approach is foreseen:

- For patients without radiation pneumonitis during study, the maximum score will be selected.
- For patients experiencing radiation pneumonitis, the score at the time of its diagnosis will be selected.

These patient-specific scores represent the fundamental units for all further statistical analyses. First of all, sensitivity and specificity will be estimated for every possible cut-off value of the scoring system. The Receiver Operating Characteristic (ROC) curve is used to show in a graphical way the relation between sensitivity and specificity. It is defined as the plot of sensitivity versus 1-specificity (false-positive rate) across varying cut-offs. A ROC curve corresponding to greater discriminant capacity of the scoring system is located closer to the upper-left-hand corner. An ROC curve lying on the diagonal line reflects the performance of a diagnostic test that is no better than chance level.

The area under the curve (AUC) summarizes the entire location of the ROC curve. The AUC is an effective and combined measure of the sensitivity and specificity that describes the inherent validity of the usefulness of the test in general, where a greater area means a more useful test. If AUC is 1, the symptom-based scoring system is perfect in the differentiation between patients with and without radiation pneumonitis. This happens when the distribution of the score values for the patients with and without events do not overlap. In contrast, AUC = 0.5 means that the scoring system is performing no better than chance. Therefore, the AUC can be considered as a valuable quantitative measure to prove the diagnostic ability of the scoring system. A rough guide for classifying the accuracy of a diagnostic test is the traditional academic point system (AUCs of 0.5–0.6 = fail; 0.6–0.7 poor; 0.7–0.8 = fair, 0.8–0.9 = good and 0.9–1 = excellent). Therefore, any symptom-based score leading to an AUC of ≤0.7 will be rated insufficiently useful.

Based on this definition, the following hypothesis system will be subjected to statistical analysis:

H0: AUC = 0.7 versus H1: AUC ≠ 0.7

Non-parametric methods for AUC estimation and testing using the normal approximation of the asymptotic properties of the AUC with standard errors derived by the method of DeLong, DeLong and Clarke-Pearson will be applied [20]. The SAS (SAS Institute Inc.) LOGISTIC procedure with the ROCCONTRAST statement can be used to estimate the AUC and its 95% confidence limit and to provide the *p*-value for the test mentioned above. A significance level of two-sided 5% is pre-specified.

If statistical significance of the AUC is reached, the most-informative (optimal) scoring point to predict radiation pneumonitis will be established. Based on discussions with experts optimality is defined as a score cut-off value with sensitivity ≥90% and specificity ≥80%. In addition to this visual selection of a suitable cut-off value, the Youden index will be applied to propose an optimal cut-off value for further consideration.

As a further sensitivity analysis the relationship between tertiles of symptom-based scores and incidence of radiation pneumonitis will be statistically tested using the Jonckheere-Terpstra test, a nonparametric test for ordered differences among groups of score values. It tests the global null hypothesis that the distribution of the response variable does not differ among tertiles. The test is designed to detect alternatives of ordered differences, meaning that the incidence of pneumonitis increases with the tertiles of score values.

For further exploratory analysis a logistic regression model will be constructed using a backward stepwise selection procedure using the individual three symptoms of the scoring system as independent (dichotomized) variables and the presence of radiation pneumonitis as dependent variable. Specific symptoms will be removed if this exclusion does not result in a significant chance in the log-likelihood ratio test. The cut-off for variable removal will be set at a significance level of 0.10. Based on the resulting model, a predictive score for clinical use will be derived by multiplying each ß coefficient by 10 and rounding to the nearest integer. The integers will be added together to produce an overall symptom-based score for each patient. To evaluate the ability of the score to predict increasing risk of radiation pneumonitis, the ROC curve will be graphically displayed and an optimal cut-off point will be selected based on the methods described above. The goal of this additional exploratory analysis is to assess whether the scoring points proposed by the expert panel for each symptom (before start of this study) can be relevantly improved by applying purely data-driven multivariable statistical methods. The derived cut-off value should be considered as a preliminary suggestion which has to be validated in subsequent studies.

#### Secondary aims

1. Positive and negative predictive values associated with each scoring point of the symptom-based scoring system. The positive predictive value is the probability that subjects with a high symptom score truly suffer from radiation pneumonitis. The negative predictive value is the probability that subjects with a low symptom score truly don’t suffer from radiation pneumonitis. Thus, the predictive values describe the performance of the scoring system and the relevance for the patients whereas sensitivity and specificity describe the intrinsic validity of the test criterion.
  Since the incidence of subjects experiencing a radiation pneumonitis to be observed in this study reflects the incidence of the target population with the specific inclusion/exclusion criteria (i.e. the number of subjects with pneumonitis is not pre-specified), the positive and negative predictive values for each potential cut-off can be estimated unbiasedly. Point estimates of positive and negative predictive values will be presented.
2. Patient satisfaction with the symptom-based scoring system (symptom-questionnaire, paper version), assessed at the end of radiotherapy. Statistical analysis consists of presenting the respective proportions. In case of a dissatisfaction rate > 20%, the scoring system needs modifications before used in future studies. In case of a dissatisfaction rate > 40%, the symptom-based scoring system is considered not useful.
3. Quality of life (QoL): Evaluation prior to radiotherapy, at the end of radiotherapy and at the end of follow up using the EORTC QLQ-C30 Version 3.0 and the EORTC QLQ-LC13 (https://qol.eortc.org). Global health status, functional scales and symptom scales/items of both instruments will be separately correlated to the scoring points of the symptom score obtained in this study.
4. Patient satisfaction with the mobile application, assessed at the end of radiotherapy. The satisfaction results will be described by means of descriptive analyses. In case of a dissatisfaction rate > 20%, the app needs modifications before it can be further investigated in future studies. In case of a dissatisfaction rate > 40%, the app will be considered not suitable for further investigation.
  Analyses are mainly performed at the end of radiotherapy, since the radiation doses at end-of-study vary between the patients included in this trial.

### Data management and monitoring

All data related to patients will be recorded in a pseudonymous way. Each patient will be identifiable only by the unique patient number, date of birth and gender. A patient identification list will only be kept in the trial centre and not be forwarded to the sponsor. All data will be pseudonymised before forwarded for analysis. The data will be handled according to the General Data Protection Regulation (GDPR). The originals of all key trial documents including documentation sheets will be kept at the trial headquarters (i.e. the responsible sponsor) for at least 10 years after the final report. The principal investigator will keep all administrative documents, patient identification list, signed informed consent forms, copies of the documentation sheets and general trial documentation. Original patient data (patient files) must be kept for the period time required at the corresponding trial centre but not for < 10 years.

The ZKS Lübeck will conduct clinical on-site monitoring according to GCP and written standard operating procedures (SOPs) to ensure the patients’ rights and safety as well as the reliability of trial results.

For initiation, the trial site will be visited on-site by a clinical research associate of the ZKS Lübeck. During the trial, the site will be visited at regular intervals depending on the rate of recruiting and data quality. Informed consent and defined key data will be checked of all patients. The medical file of each patient will be screened for adverse and serious adverse events. Patients’ questionnaires will be checked for their existence. According to SOPs, all trial specific monitoring activities will be defined before starting the trial and documented in writing (monitoring manual). No regular audits are planned. However, to ensure correct execution of the study, audits may be conducted if necessary. As the current study is not related to the German pharmaceutical or medicinal product act, no inspections of higher federal authorities are scheduled.

Moreover, a data monitoring committee is not required, since all patients participating in this trial receive the same cancer treatment and the same treatment for radiation pneumonitis and other toxicities as they would have received if not participating in the trial.

The coordinating investigator will work towards comprehensive internal and external dissemination of project results and knowledge. Coordinating investigator, biostatistician and staff members of the center where the study is performed will create a report regardless of regular or abnormal study termination. The scientific results will be published in an international, peer-reviewed journal. In addition, results are planned to be presented at meetings and symposia. All reports and publication related to the study need to be coordinated with the biostatistician to avoid misinterpretations. Conclusions need to be statistically secured and require approval of the statistician. For publications of any kind the study acronym PARALUC will be used. Data analysts and statisticians are blinded to assure anonymization (data protection).

Amendments to the study protocol may only be implemented if again approved by the responsible ethics committee. Only the coordinating principal investigator may carry out such changes. However, all co-investigators should contact the coordinating principal investigator if modifications seem to be necessary. In case of changes to the study protocol, all investigators will be informed after ethics committee approval and the notice has to be confirmed.

## Discussion

Radiation pneumonitis is a serious adverse event in patients irradiated for lung cancer. It was reported to result in event-related death in about 2% of the affected patients [3]. In a previous retrospective study of 256 lung cancer patients, the 3-year survival rates of patients with no (*n* = 162), mild (*n* = 69) and severe (*n* = 25) radiation pneumonitis were 33, 38 and 0%, respectively [21]. Radiation pneumonitis may develop up to several months following treatment [4]. Symptoms such as cough, shortness of breath and fever may not be associated with the radiation treatment that took place several weeks ago. Thus, radiation pneumonitis can be missed, and patients are often treated with antibiotics alone for bronchitis or pneumonitis, which is not effective for radiation pneumonitis. As a consequence, the adverse event often becomes more severe before it is eventually diagnosed [5].

Therefore, it is very important to be able to have an instrument that helps the treating physicians identify radiation pneumonitis early. The PARALUC trial has been designed to contribute to such an instrument. It is performed in patients with a comparably high risk of radiation pneumonitis, since certain numbers of events are required to develop the scoring system [4]. For the creation of a symptom-based scoring system, the patients are asked to complete a paper-based questionnaire once a week and rate the three main symptoms of pneumonitis, namely cough, shortness of breath and fever. Scoring points are assigned to the severity of the symptoms, and the resulting sum scores are used to identify radiation pneumonitis. The main goal of this study is to establish the most-informative (optimal) scoring point to predict radiation pneumonitis. Based on discussions with experts, optimality has been defined as a sum score (cut-off value) achieving a sensitivity of ≥90% and a specificity of ≥80%. Moreover, in 10 patients the paper-based questionnaire is supplemented by an app that asks the same questions regarding the main symptoms of pneumonitis. These patients are asked about their satisfaction with the app. For the development of such an app, the new scoring system is more suitable than existing tools such as the CTCAE and CTCAE-PRO [6, 22, 23] (https://healthcaredelivery.cancer.gov/pro-ctcae/pro-ctcae_german.pdf). The app is intended to be used by the patients at home to rate their symptoms daily and allow fast intervention if necessary. The CTCAE-PRO is related to symptoms the patients experienced during the last 7 days and, therefore, does not represent the current situation on a specific day as required for the app [22, 23] (https://healthcaredelivery.cancer.gov/pro-ctcae/pro-ctcae_german.pdf). Moreover, the wording of the CTCAE-PRO is less precise compared to the scoring system of the present study. Also the CTCAE is not suitable for the app, since it includes objective assessments by medical staff members including the need for medical interventions and does not focus on the self-rating of symptoms by the patients [6]. One has to be aware that with a symptom-based scoring system, grade 1 pneumonitis will be missed, since it is defined as asymptomatic according to CTCAE v5.0 [6]. However, this limitation may not be clinically important, since medical intervention is not indicated for grade 1 pneumonitis [6]. If radiation pneumonitis becomes symptomatic, it should be identified by a symptom-based score. It will be investigated in the PARALUC trial, whether this assumption is correct.

If in the PARALUC trial, an optimal cut-off score resulting in sufficiently high sensitivity and specificity can be identified and if the patients are sufficiently satisfied with the functionality and practicability of the app in its current form, a future step will be the development of an app that can be used by the patients at home. Both, the symptom-based scoring system created in the PARALUC trial and the future app will contribute to the identification of radiation pneumonitis and likely lead to an improvement of the prognoses of patients irradiated for lung cancer.

## Data Availability

Not applicable, as no datasets were generated or analysed during the current study so far. The study has been registered at clinicaltrials.gov (identifier: NCT04335409), where data regarding this study are available.

## Abbreviations

AUC: Area under the Curve
CTCAE: Common Terminology Criteria for Adverse Events
DLCO: Diffusing Capacity of the Lung for Carbon Monoxide
EORTC: European Organisation for Research and Treatment of Cancer
FEV1: Forced Expiratory Volume in 1 Second
GCP: Good Clinical Practice
PARALUC: Pneumonitis After RAdiotherapy for LUng Cancer
QLQ: Quality of Life Questionnaire
ROC: Receiver Operating Characteristic
SOP: Standard Operating Procedure
VMAT: Volumetric Modulated Arc Therapy
